# Localization of P2X receptor subtypes 2, 3 and 7 in human urinary bladder

**DOI:** 10.1186/s12894-015-0075-9

**Published:** 2015-08-08

**Authors:** Karl Svennersten, Katarina Hallén-Grufman, Petra J. de Verdier, N. Peter Wiklund, Mirjana Poljakovic

**Affiliations:** Department of Molecular Medicine and Surgery, Section of Urology, Karolinska Institutet, 171 76 Stockholm, Sweden; Department of Urology, Karolinska University Hospital, Stockholm, Sweden; Department of Laboratory Medicine, Karolinska Institutet, Stockholm, Sweden

## Abstract

**Background:**

Voiding dysfunctions are a common problem that has a severe negative impact on the quality of life. Today there is a need for new drug targets for these conditions. The role of ATP receptors in bladder physiology has been studied for some time, primarily in animal models. The aim of this work is to investigate the localization of the ATP receptors P2X2, P2X3 and P2X7 and their colocalization with vimentin and actin in the human urinary bladder.

**Methods:**

Immunohistochemical analysis was conducted on full-thickness bladder tissues from fundus and trigonum collected from 15 patients undergoing open radical cystectomy due to chronic cystitis, bladder cancer or locally advanced prostate cancer. Colocalization analyses were performed between the three different P2X subtypes and the structural proteins vimentin and actin. Specimens were examined using epifluorescence microscopy and correlation coefficients were calculated for each costaining as well as the mean distance from the laminin positive basal side of the urothelium to the vimentin positive cells located in the suburothelium.

**Results:**

P2X2 was expressed in vimentin positive cells located in the suburothelium. Less distinct labelling of P2X2 was also observed in actin positive smooth muscle cells and in the urothelium. P2X3 was expressed in vimentin positive cells surrounding the smooth muscle, and in vimentin positive cells located in the suburothelium. Weaker P2X3 labelling was seen in the urothelium. P2X7 was expressed in the smooth muscle cells and the urothelium. In the suburothelium, cells double positive for P2X2 and vimentin where located closer to the urothelium while cells double positive for P2X3 and vimentin where located further from the urothelium.

**Conclusion:**

The results from this study demonstrate that there is a significant difference in the expression of the purinergic P2X2, P2X3 and P2X7 receptors in the different histological layers of the human urinary bladder.

## Background

Functional bladder disturbances are very common and have a negative impact on the quality of life for the affected individuals. Today, disturbances in the filling and voiding of the urinary bladder are primarily treated with substances interfering with the cholinergic signalling system, i.e. antimuscarinic drugs. These drugs are initially effective, but have adverse effects that are often associated with poor compliance and persistence with treatment [1]. Therefore, it is highly relevant to investigate alternative targets. Forty years ago, purinergic receptors were suggested to be an important component of the non-adrenergic, non-cholinergic (NANC) transmission in the urinary bladder [2], and ever since, there have been several reports on the importance of the purinergic signalling system in the urinary bladder based on both functional and histological studies [3–8].

Purinergic signalling is mediated via the release of ATP and its metabolites. The P2X receptors are ion-channels [9, 10] and there are several reports on these receptors being present in the urinary bladders of different species [7, 11–13]. However, most studies performed on human urinary bladders have investigated individual cell types [5–7] or performed quantitative PCR analysis of whole urinary bladders [14].

Several different P2X subunits have been described in the urinary bladder. In the present work we have limited ourselves to investigate the distribution of P2X2, P2X3 and P2X7 subunits in relation to actin and vimentin in the human urinary bladder. We have chosen to look at P2X2, P2X3 and P2X7 since they have different pharmacological properties. P2X2 is slowly desensitizing, P2X3 is fast desensitizing and P2X7 is non-desensitizing [15]. P2X2 and P2X3 are believed to be involved in the neuroeffector unit [16] and P2X7 has been shown to be involved in inflammatory responses [17] as well as in actin reorganisation [18]. Even though the P2X3 receptor has been found both on interstitial cells (ICs) and afferent nerve endings in the human urinary bladder its expression in the smooth muscle layer has not been fully investigated [6]. P2X2 is commonly expressed together with P2X3 in P2X2/3 heterotrimers and in this regard it is interesting to compare the distribution of these subtypes [10].

## Materials and methods

### Tissue

All experiments presented in this paper were approved by the Regional Ethics Committee at Karolinska University Hospital (Dnr. 2010/574-32, 2009/1481-32, 2008/1633-31) and all samples were collected from patients after retrieval of written informed consent according to the declaration of Helsinki.

Human urinary bladder tissue was collected from 6 male and 9 female patients (age range 58–82 years, mean age 72 years) undergoing open radical cystectomy due to chronic cystitis, bladder cancer or locally advanced prostate cancer. All patients were screened for ongoing urinary tract infection prior to surgery. All patients received prophylactic perioperative antibiotic treatment. Patients were also screened for comorbidities that could pose as contraindications for the surgery such as disseminated malignant disease or severe heart failure. All collected tissue was fixed in 4 % formaldehyde (Apoteket, Gothenburg, Sweden) , cryoprotected before frozen in Tissue Tek O.C.T. (Sakura Finetek Sweden AB, Gothenburg, Sweden) and sectioned in 10 μm sections using a MICROM HM 525 cryostat (MICROM International GmbH, Walldorf, Germany).Sampled tissues from the bladder trigonum and fundus were all taken from macroscopically tumor free areas, as determined by the pathologists at the Department of Pathology and Oncology and encompassed the full bladder thickness (urothelium, submucosa/lamina propria, smooth muscle, and serosa). All bladders were investigated microscopically by a pathologist and are matched with a pathology report verifying the diagnosis.

### Immunofluorescence

Slides were pre-incubated in PBS containing 0.05 % Triton X-100 and 0.2 % bovine serum albumin (BSA; both from Sigma-Aldrich, St Louis, USA). Slides were then incubated with primary antibodies; rabbit anti-P2X2 (1:50; H-116; Santa Cruz Biotechnology Cat# sc-25693, RRID:AB_2157914) [19], rabbit anti-P2X3 (1:50; H-60; Santa Cruz Biotechnology Cat# sc-25694, RRID:AB_2158071) [19, 20], rabbit anti-P2X7 (1:50; H-265; Santa Cruz Biotechnology Cat# sc-25698, RRID:AB_2158373) [21], goat anti-vimentin (1:250; C-20; Santa Cruz Biotechnology Cat# sc-7557, RRID:AB_793998) [22] and rabbit anti-laminin (1:200; Sigma-Aldrich Cat# L9393, RRID:AB_477163) [23] diluted in PBS containing 0.05 % Triton X-100 and 0.2 % BSA over-night in a moist chamber at room temperature. All antibodies were raised against human antigens except anti-laminin which was raised against basement membrane of Englebreth Holm-Swarm (EHS) mouse sarcoma. After washing in PBS, the slides were incubated in different mixtures of species-specific goat or donkey secondary antibodies conjugated with Dylight488 (1:500; Jackson ImmunoResearch Laboratories, Inc, West Grove, USA), Alexa Fluor 568, Alexa Fluor 488 (1:200; Lifetechnology, Carlsbad, USA) and/or actin-binding TRITC-phalloidin (1 μg/ml; Sigma-Aldrich) for one hour at room temperature in a moist chamber. Slides were then rinsed in PBS before mounted with Dako Fluorescence Medium (DakoCytomation, Glostrup, Denmark) to prevent fading. Secondary antibody specificity was assessed by omitting the primary antibody.

### Immunofluorescence microscopy and image analyses

Immunofluorescence image acquisition was performed using a Nikon Eclipse 800 fluorescence microscope equipped with a Nikon DXM1200 digital camera and Nikon ACT-1 version 2.10 software. Image analysis was conducted using Image J (U. S. National Institutes of Health). Mean distance was calculated by measuring the distance from the border between urothelium and suburothelium to three different cells per image, which labelled positive for both vimentin and P2X2 or P2X3 respectively. The border between the suburothelium and the urothelium was verified by immunoreactivity to laminin in the basal membrane and could be identified both in vimentin and P2X labelled images. The mean distance for these cells was calculated for each image. Images were assembled using the free- and open source program GIMP (GNU Image Manipulation Program)2.6.11 developed by The GIMP Development Team freely available at www.gimp.org. Schematic drawing was created using the free- and open source program Inkscape 0.48 developed by The Inkscape Team freely available at inkscape.org.

### Analysis of data

Results from colocalization analysis are expressed with Pearson’s correlation coefficient and Manders’ overlap coefficient as described by Manders et al. [24]. Pearson’s correlation coefficient was calculated as

$$ {r}_p=\frac{{\displaystyle {\sum}_i\left({R}_i-\overline{R}\right)\cdot \left({G}_i-\overline{G}\right)}}{\sqrt{{\displaystyle {\sum}_i{\left({R}_i-\overline{R}\right)}^2\cdot {\displaystyle {\sum}_i{\left({G}_i-\overline{G}\right)}^2}}}} $$

and the Manders’ overlap coefficient was calculated as

$$ {r}_m=\frac{{\displaystyle {\sum}_i{R}_i\cdot {G}_i}}{\sqrt{{\displaystyle {\sum}_i{\left({R}_i\right)}^2\cdot {\displaystyle {\sum}_i{\left({G}_i\right)}^2}}}} $$

R_i_ and G_i_ are grey values of pixel i from the red and green channel of a dual-color image. 8-bit grey scale images acquired from the red and green channel was correlated with each other. Full scale uncropped images were used for all analysis except in Fig. 4 where the cells of interest were outlined using the Image J ROI-tool before analysis. Mean and standard error of the mean (SEM) of the Pearson’s correlation coefficient and Manders’ overlap coefficient were calculated for the different combinations of costaining. To test for significant differences in colocalization, the Student’s unpaired t-test was used to compare two means and ANOVA followed by Bonferroni post-hoc test was used for multiple comparisons (Microsoft Excel with ad-in Data Analysis) as recommended by McDonald et al. [25]. A p-value < 0.05 was considered statistically significant. Pearson’s correlation coefficient and Manders’ overlap coefficient was calculated using the Image J plugin colocalization finder (developed by Cristophe Laummonerie and Jerome Mutterer at Institut de Biologie Moleculaire des Plantes, Strasbourg, France) and Intensity correlation analysis (from Wright cell imaging facility, www.uhnres.utoronto.ca/facilities/wcif/). No image processing or background subtraction was performed before calculation of Pearson’s correlation coefficient. Before calculation of Manders’ overlap coefficient, the background of all images was subtracted using Image J plugin ROI background subtract. Graph-plotting was conducted using Microsoft Excel.

## Results

### P2X expression in the human urinary bladder smooth muscle layer

**Fig. 1:**
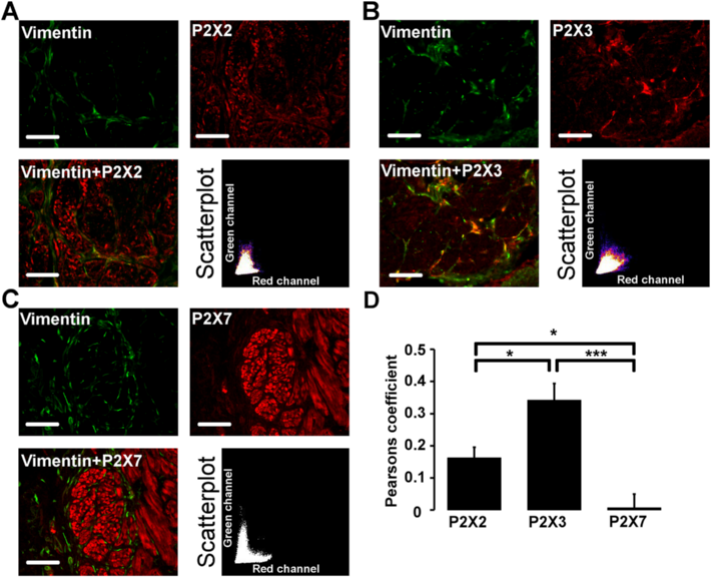
Vimentin and P2X receptor immunostaining in human urinary bladder smooth muscle layer. **a-c**. Costaining (yellow) of the smooth muscle layer with vimentin (green) and P2X (red), including scatterplot of red and green pixel intensities. Sections are from a patient with bladder cancer. **d.** Pearson’s correlation coefficient for vimentin (green channel) and P2X (red channel) expressed as mean ± SEM (P2X2: *n* = 11, P2X3: *n* = 15 and P2X7: *n* = 14). *p < 0.017 and ***p < 0.00017 for Bonferroni adjusted levels of significance. Scale bar = 100 μm

The immunoreactivity for the P2X2 receptor in the smooth muscle layer of the human urinary bladder was moderate. The predominant P2X2 immunoreactivity was observed in smooth muscle cells and in vimentin positive cells located in the periphery, as well as, in between the smooth muscle bundles (Fig. 1a). P2X3 immunoreactivity was present in vimentin positive cells but absent in the smooth muscle cells (Fig. 1b). P2X7 immunoreactivity was seen in the smooth muscle cells. No P2X7 labelling was observed in vimentin positive cells located in the muscle layer. This observation was confirmed by the low correlation between vimentin and P2X7 immunoreactivity in the smooth muscle layer of the human urinary bladder (Fig. 1c-d). By calculating the Pearson´s correlation coefficient between vimentin and the different receptor subtypes, we observed that there was a significant variation between the different P2X receptors analysed with the relation P2X3 > P2X2> > P2X7 with regards to colocalisation with vimentin (Fig. 1d). This relationship was also present when data from the trigonum and the fundus of the bladder were analysed separately but there were no significant differences in the Pearson´s correlation coefficient between trigonum and fundus for the respective P2X receptors. Also, by using Manders’ overlap coefficient analysis, a significant difference in colocalization with vimentin between P2X3 and P2X7 was obtained (Table 1).

**Table 1:** Manders’ overlap coefficient for P2X in smooth muscle layer (mean ± SEM)

	P2X3	P2X7	*p*-value
Vimentin	0.69 ± 0.03	0.43 ± 0.03	1.63*10^−5^
Actin	0.53 ± 0.04	0.86 ± 0.03	6.07*10^−7^

**Fig. 2:**
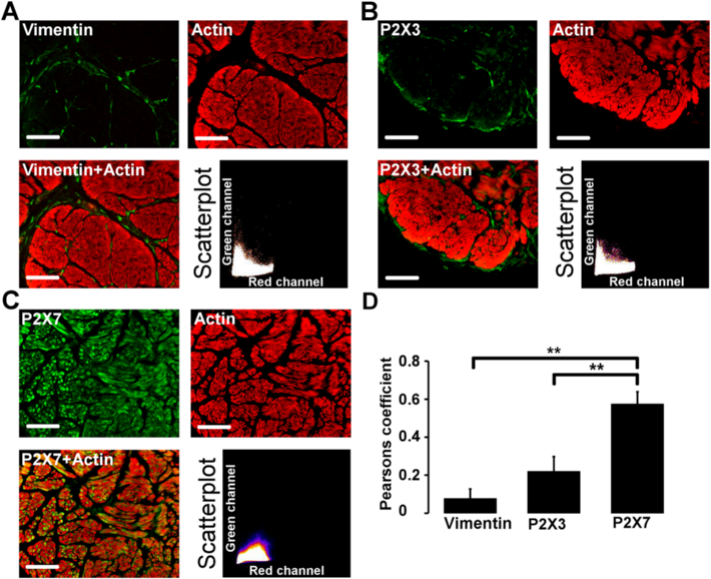
Actin and vimentin or P2X receptor immunostaining in human urinary bladder smooth muscle layer. **a**. Costaining (yellow) of the smooth muscle layer with actin (red) and vimentin (green), with scatterplot of red and green pixel intensities. Section is from a patient with prostate cancer. **b-c**. Smooth muscle layer double labelled with P2X (green) and actin (red) with scatterplot of red and green pixel intensities. Sections are from a patient with bladder cancer. **d**. Pearson’s correlation coefficient for actin (red channel) and P2X/vimentin (green channel) expressed as mean ± SEM (*n* = 9). *p < 0.017 and **p < 0.0017 for Bonferroni adjusted levels of significance. Scale bar = 100 μm

In order to distinguish between the smooth muscle ICs and muscle cells, which showed immunoreactivity for P2X3 and P2X7 respectively, the bladder tissues were costained for vimentin and actin filaments using anti-vimentin antibodies and TRITC-conjugated phalloidin, respectively. The smooth muscle bundles were intensely labelled by the phalloidin, revealing high actin content, while actin labelling of the vimentin positive ICs was negligible as compared to actin labelling of the smooth muscle. This is illustrated by the low correlation between the labelling of vimentin and actin (Fig. 2a, d). Subsequent costaining of P2X3 with TRITC-phalloidin showed that the immunoreactivity of the P2X3 in vimentin positive ICs was significantly separated from the phalloidin labelled smooth muscle (Fig. 2b, d). The P2X7 receptor subtype and actin were both intensely labelled and their localization in the muscle bundles correlated well with each other (Fig. 2c-d). The Pearson’s-correlation coefficient for P2X7 and actin was significantly higher than for P2X3 and actin as well as for actin and vimentin (Fig. 2d). Also, by using Manders’ overlap coefficient a significant difference in colocalization with actin between P2X7 and P2X3 was seen (Table 1).

### P2X expression in the human urinary bladder urothelium and suburothelium

**Fig. 3:**
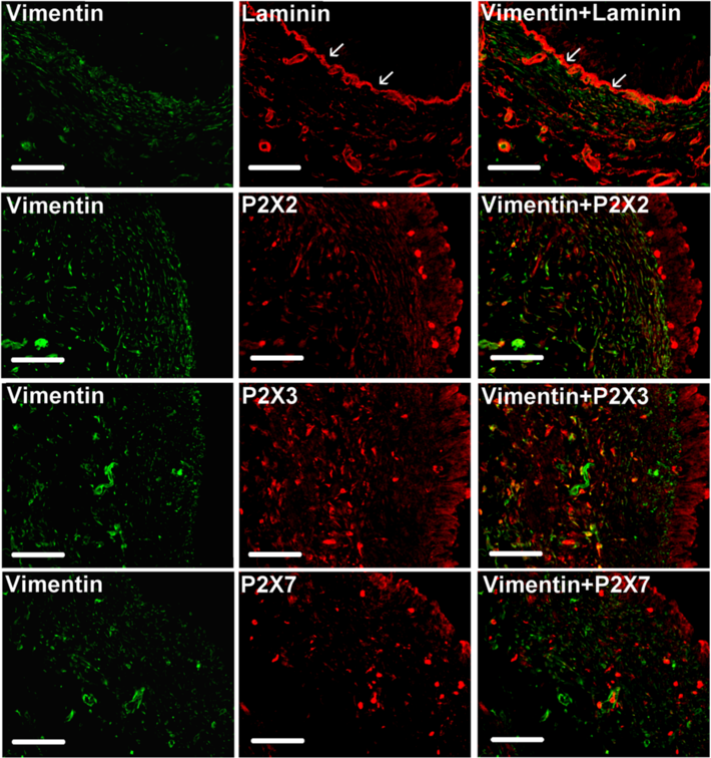
Laminin, vimentin and P2X receptor immunostaining in human urinary bladder urothelium and suburothelium. Top panel; Double immunostaining of vimentin (green) and laminin (red). Arrows indicate anti-laminin labelling of the basal membrane. Section is from a patient with prostate cancer. Scale bar = 100 μm. Following panels; Double immunostaining of vimentin (green) and P2X2, P2X3, and P2X7, respectively (red). Yellow indicates double immunoreactivity to vimentin and P2X. Sections are from a patient with bladder cancer. Scale bar = 100 μm

**Fig. 4:**
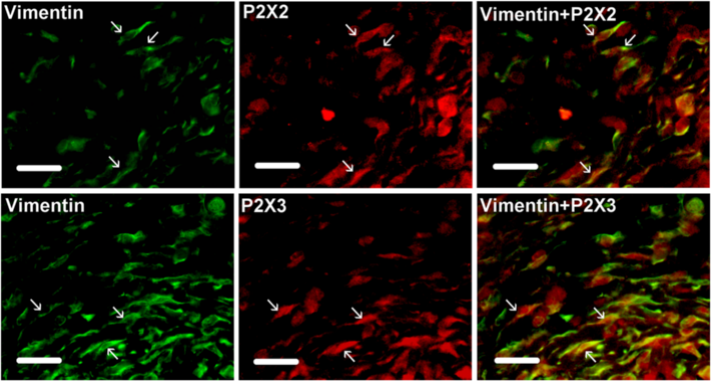
Top panel; Immunohistochemical labeling of vimentin (green) and P2X2 (red) in human urinary bladder suburothelium. Yellow = immunoreactivity to both vimentin and P2X2. Arrows indicate cells positive for vimentin and P2X2. Manders’ overlap coefficient for cells indicated by arrows = 0.93 ± 0.01 (mean ± SEM). Sections are from a patient with prostate cancer. Scale bar = 25 μm. Bottom panel; Immunohistochemical labeling of vimentin (green) and P2X3 (red) in human urinary bladder suburothelium. Yellow = immunoreactivity to both vimentin and P2X3. Arrows indicate cells positive for vimentin and P2X3. Manders’ overlap coefficient for cells indicated by arrows = 0.87 ± 0.04 (mean ± SEM). Sections are from a patient with prostate cancer. Scale bar = 25 μm

**Fig. 5:**
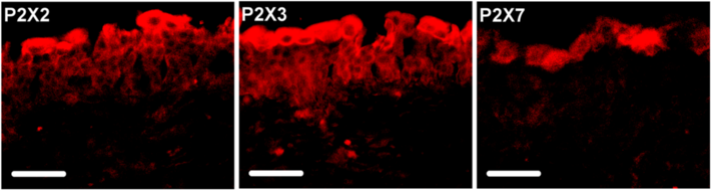
Immunohistochemical labeling of purinergic receptors (red) in human urinary bladder urothelium, from left to right: P2X2, P2X3 and P2X7. Sections are from a patient with prostate cancer. Scale bar = 50 μm

The border between urothelium and suburothelium was visualised using polyclonal antibodies against laminin which is abundant in the basal membrane of the human bladder (Fig. 3). P2X2 immunoreactivity was observed in vimentin positive cells in the urinary bladder suburothelium verified by calculating Manders’ overlap coefficient for single cells (Figs. 3 and 4). P2X2 was also observed in the urothelium, primarily in apical cells and to a lesser extent in urothelial cells located in the basal and intermediate layer of the urothelium (Fig. 3 and 5). Abundant P2X3 receptor immunoreactivity was seen in vimentin positive cells and in the urothelium (Figs. 3, 4 and 5). The P2X7 receptor immunoreactivity was absent in vimentin positive cells but present in apical cells in the bladder urothelium (Figs. 3 and 5).

The P2X3 immunoreactivity was commonly more intense compared to P2X2 and P2X7 in vimentin positive cells located in the suburothelium. The intensity of the labelling of vimentin positive cells followed the order P2X3 > P2X2 > P2X7 and were significantly different from each other (normalised intensity was 1.53 ± 0.07 for P2X3, 1.29 ± 0.03 for P2X2 and 1.16 ± 0.02 for P2X7). There was no significant difference in the intensity of the labelling of the purinergic receptors between the trigonum and the fundus of the bladder. The distribution of vimentin positive cells in the suburothelium was heterogenous with a dense layer of vimentin positive cells close to the urothelium and an area of more sparsely distributed vimentin positive cells closer to the smooth muscle. Also, the location of cells positive for the different P2X receptors varied in the suburothelium. By measuring the distance from the basal side of the urothelium to the vimentin positive cells that were most intensely labelled for the respective P2X receptors, we found that the most intense P2X2 labelling was on average 73 ± 14 μm from the urothelium while P2X3 was 186 ± 25 μm from the urothelium. Generally, there was a greater distance between vimentin positive cells and the urothelium in the trigonum than in the fundus but this difference was not significant.

## Discussion

**Fig. 6:**
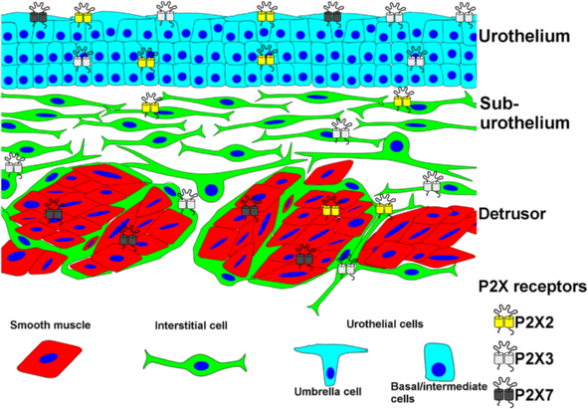
Scheme depicting the distribution of the investigated P2X subunits in the different histological areas of the human urinary bladder

We have used antibodies against the purinergic receptor subunits P2X2, P2X3 and P2X7 to show that these receptor subunits are expressed differently throughout the histological layers of the human urinary bladder. P2X2 is present in vimentin positive cells of the suburothelium and in the vicinity of the smooth muscle bundles, and to some extent in the smooth muscle and urothelium. P2X3 is found in the urothelium and in vimentin positive cells located in the suburothelium as well as in vimentin positive cells surrounding smooth muscle bundles. The expression of P2X7 is predominantly localized to the smooth muscle cells of the human urinary bladder but also to some extent in the apical cells of the urothelium (Fig. 6). Hence, P2X2, P2X3 and P2X7 receptors might have different roles in the different histological parts of the urinary bladder.

The variation in expression of the different P2X receptor subunits in the different layers of the urinary bladder is interesting, since these receptors may have a role as mechanoreceptors when they respond to autocrine ATP signalling due to mechanical stress. The suburothelium of the urinary bladder is rich in ICs and these are suggested to play an important role in the transduction of mechanical input from the urothelium to the detrusor muscle. ICs in the urinary tract have been found to modulate the excitability of smooth muscle cells upon nerve stimulation [26] and to be of importance for contractile responses [27]. Interstitial cells are of mesenchymal origin and vimentin has extensively been used as a marker for ICs in the urinary bladder [6, 11, 28]. However, vimentin has also been suggested to be important in cellular mechanical stability, migration and contractile capacity [29] and it has been demonstrated that ATP can induce vimentin reorganization [30]. In our study, we have observed both P2X2 as well as P2X3 immunoreactivity in vimentin positive cells in the suburothelium of the human urinary bladder. However, the localization of P2X2 and P2X3 expression in vimentin positive cells varied in relation to the distance from the urothelium. P2X2 was generally located closer to the urothelium while P2X3 was on average located closer to the smooth muscle. In agreement with a current study [31] our results show that vimentin positive cells have different purinergic immunoreactive phenotypes based on their location in the bladder suburothelium. In addition, since both P2X2 and P2X3 immunoreactivity was observed in vimentin positive cells located in the smooth muscle layer, our results further suggest that vimentin positive cells vary phenotypically based on their histological location in the urinary bladder.

In the current study, P2X2 and particularly P2X7 were expressed in actin positive detrusor smooth muscle cells. The role of P2X7 in the actin rich muscle cell layer is especially intriguing since ATP binding to P2X7 can cause actin cytoskeleton reorganization with subsequent cellular signaling [6] and release of cytokines in inflammatory cells [18]. These findings are interesting with regard to our study because P2X7 have a role in inflammation in general and functional bladder disabilities often have an inflammatory component. This may suggest a connection between symptoms from the bladder and pathologies with an underlying inflammatory component such as the metabolic syndrome [32].

The human bladder urothelium forms a tight barrier between the lumen and the internal milieu of the urinary bladder. It also plays an important role in bladder mechanosensation with both autocrine and paracrine signalling functions [6]. The human bladder urothelium is a stratified epithelium comprising highly specialised apical cells, the umbrella cells. Recently, there have been several interesting reports on the organisation and development of the urothelial cells [33]. These reports suggest that the development of the different layers in the stratified urothelium is not a continuous process, but rather that the umbrella cells have their own lineage originating from a subclass of cells in the basal layer. In the current study we observed that all three investigated P2X receptors are expressed in the umbrella cells. Furthermore, P2X3, and to a lesser extent, P2X2 immunoreactivity was observed in the entire human urothelium. Due to the cellular complexity of the urothelium there are conflicting reports related to protein expression in the urothelium particularly, the expression of the purinergic receptors (reviewed in [34]). In the current study, no immunoreactivity in the human urothelium was obtained when the tissue sections were incubated with the secondary antibody alone. Indeed, this control for antibody specificity has been questioned but P2X2 and P2X3 has previously been demonstrated in human urothelium [35] and P2X7 in the rat urothelium [36].

The organized distribution of different P2X subtypes in the human urinary bladder is of clinical and pharmacological significance. This is especially intriguing, since there are interesting results from animal studies that show that the increased micturition frequency seen in older animals is accompanied with an increase of purinergic signalling and a decrease of cholinergic [37]. There are also indications that anticholinergic treatment of overactive bladder syndrome may shift the balance between purinergic and cholinergic transmission towards more of purinergic [38]. This could explain increasing therapy unresponsiveness over time.

Results from these studies as well as the finding of organized expression of P2X receptors in human bladders from older patients suggest that the purinergic system may be a better target if one wants to treat lower urinary tract symptoms (LUTS). What is promising with our findings is that there are a number of specific agonists against specific P2X receptors [39] which raise the hope of finding efficient drugs with few side effects. The results from our study is highly relevant for the potential treatment of LUTS since the bladders used in our study are from older men and women undergoing cystectomy by various pathologies associated with LUTS.

A limitation with this study is that we have not been able to use urinary bladders from healthy individuals. The urinary bladders are from patients undergoing radical cystectomy due to bladder cancer, chronic cystitis or locally advanced prostate cancer. The dissected specimens have been free of tumour both macro- and microscopically, but the disease itself can affect local and systemic physiology. For example, P2X7 expression has been found to be increased in non-tumour cells in the vicinity of malignant cells in prostate cancer [40] while the expression of P2X3 in human bladder urothelium decreases with increased grade of malignancy [41]. Tissue hosting tumours may also have alterations on a molecular level even though they have normal histology [42]. However, in our study we did not see any difference in P2X expression between bladders that had been removed due to different diagnosis. Also, our results are comparable to studies performed on healthy animal bladders [36], leading us to interpret purinergic signalling as a general component of bladder physiology present in unaffected bladders as well as those affected by disease.

## Conclusions

In this study we show that there is a significant difference in the expression of the purinergic P2X2, P2X3 and P2X7 receptors in the different histological layers of the human urinary bladder. P2X7 is expressed in smooth muscle, P2X2 in suburothelial cells located closer to the urothelium, P2X3 in suburothelial cells closer to the smooth muscle layer and in ICs surrounding muscle bundles, while all three receptors where expressed in the urothelium. The organized distribution of different P2X receptors in the bladder supports the idea that specific targeting of the different receptors can have different effects on bladder function.

## Abbreviations

ANOVA: Analysis of variance
IC: Interstitial cell
LUTS: Lower urinary tract symtoms
NANC: Non-adrenergic, non-cholinergic
PBS: Phosphate buffered saline
ROI: Region of interest
